# Department of Canine, Feline, and Avian Medicine and Surgery

**Published:** 1901-05

**Authors:** Cecil French

**Affiliations:** Washington, D. C.


### DEPARTMENT OF CANINE, FELINE, AND AVIAN MEDICINE AND SURGERY.

By Cecil French, D.V.S.,
WASHINGTON, D. C.

Multiple Pure Lipomata in a Parrot. The literature on the subject of neoplasia among aves is scant. This fact is attributable partly to the rarity with which instances are brought to the notice of the profession, and partly because interested and willing investigators are few and far between. Hence a report on a tumor formation in a parrot, which recently came to my notice and which proved to be a pure lipoma, cannot fail to be of interest from a scientific stand-point. In any case pure lipomata are rare. Most frequently they are found associated with other tissue elements, occurring as fibrolipoma, myxolipoma, etc. J. Bland Sutton, the learned British comparative pathologist, in his *Introduction of General Pathology*, notes that lipomata occasionally occur in domestic animals, but are excessively rare in wild ones, even those born in captivity showing no exception to the rule. With regard to the frequency of their occurrence in domestic animals, Fröhner (*Monatshefte für prakt. Thierheilk.*, 1894, vi., i., p. 7) places their percentage in dogs at 6 to 7, as compared with other neoplasms, and we are all familiar with their not infrequent occurrence in horses, cattle, and sheep.

Parrots are wild animals, always being captured from the nest when very young. Therefore, though they are very commonly domesticated, yet they cannot be classed as domestic.

The bird in question is one of the Mexican breed, and of unknown age, but probably a good way on in life, as it is very tame and highly educated. I found the growths situated as follows: One, which was the first to make its appearance, was on the breast and about the size of a quarter of a dollar. The next to appear was of similar dimensions, and rose on the left wing almost at the extremity of the fleshy portion. Another smaller one appeared on the right side of the face, and about the same time still another on the left leg. All developed within a period of twelve months.

The growths were soft with intact surface, excepting the one on the wing, which had been rendered sore by the bird pecking at it. The latter still bore one or two immature feathers, but the rest were bare. I removed the growth from the wing and submitted it to Prof. Adami, of the McGill pathological laboratory, who informed me of its histological character.

I have since communicated with Dr. Frank Baker, superintendent of the National Zoological Park, on the subject, and he informs me that he some time ago extirpated a lipomatous tumor of considerable size from near the elbow of an old Amazona which had probably been in captivity a long time. The growth was not examined microscopically, but he had no doubt of its being a lipoma.

WANTED: A KNOWLEDGE OF COMPARATIVE ANATOMY. The following extract is from an article written by a well-known human surgeon to record his investigations on experimental surgery of the ureters, and which appeared in the *Annals of Surgery*, 1892.

(Subject, a bitch.) . . . . "We had considerable trouble with the Fallopian tubes, which is always the case in making this operation" (ureterorectal anastomosis) "in the female dog, as the ureters pass over the Fallopian tubes when the dog is standing, and, consequently, when lying on its back in an operation, they are under, and we are obliged to cut through the peritoneal folds of the Fallopian tubes in order to bring the ureters through them into the rectum. . . . In the canine it must be rememberd that the Fallopian tubes are very long, and frequently extend more than halfway from the bladder to the stomach, and consequently are always in the way when making this operation!" Comment is unnecessary.

